# Promoting workplace psychological wellbeing through Yoga and Tai Chi classes in female university employees

**DOI:** 10.3389/fpsyg.2024.1502426

**Published:** 2024-12-12

**Authors:** Alice Valdesalici, Silvia Cerea, Alessandra Pecunioso, Antonio Paoli, Gioia Grigolin, Rosa Nardelli, Alessandra Armenti, Marta Ghisi

**Affiliations:** ^1^Department of General Psychology, University of Padova, Padova, Italy; ^2^Department of Biomedical Sciences, University of Padova, Padova, Italy; ^3^Communication and Marketing Area, University of Padova, Padova, Italy; ^4^U.O.C. Hospital Psychology, University-Hospital of Padova, Padova, Italy

**Keywords:** workplace, Yoga, Tai Chi, wellbeing, health promotion, interventions

## Abstract

**Introduction:**

Academic environments are known for their high demands, often resulting in significant distress among employees. Thus, identifying effective intervention strategies to mitigate workplace stress is essential. The present study aims to evaluate the potential benefits of mind–body interventions (i.e., Yoga and Tai Chi) on the psychological wellbeing and perceived mental and physical health of female university employees.

**Methods:**

A total of 166 female university employees and faculty members participated in 10 sessions of Tai Chi or Yoga. They completed self-report questionnaires assessing ruminative thoughts, somatic anxiety, general distress, perceived physical and mental health, and assertive and cooperative behaviors before and after the 10 Tai Chi/Yoga sessions. Additionally, participants completed a state anxiety questionnaire before and after Tai Chi/Yoga second and second-to-last lessons.

**Results:**

Results showed significant differences between scores pre and post Tai Chi and Yoga programs, with lower ruminative thoughts (*p* = 0.007), lower somatic anxiety (*p* < 0.001), and higher perceived mental health (*p* = 0.038) at the end of the programs (i.e., after 10 sessions) compared to the beginning. Moreover, significant differences were found in state anxiety scores, with a reduction in state anxiety at the end of the second (*p* < 0.001) and second-to-last (*p* < 0.001) lessons compared to the start.

**Conclusion:**

Our findings highlight the potential positive impact of Tai Chi and Yoga programs on the psychological wellbeing and perceived mental health of female university employees. Immediate reductions in state anxiety following single sessions further underscore the potential of these practices for short-term stress relief. Overall, the results support the implementation of mind–body practices in workplace settings to promote a healthier work environment.

## 1 Introduction

Workplaces are commonly recognized as environments that can contribute to employee stress, sometimes due to challenging conditions or obstacles, which may affect mental and physical health (Hilmiana and Alviani, 2023; Martins et al., 2023).In this perspective, companies and organizations are increasingly recognizing the importance of implementing strategies to promote the wellbeing of their workforce (Abraham, 2019; Hilmiana and Alviani, 2023).

A range of workplace health promotion interventions are currently available, with physical activity and exercise among the most common (Malik et al., 2014). Regular physical activity is widely recognized not only for its benefits in enhancing physical health (Warburton et al., 2006) and lowering the risk of various chronic diseases (Nyberg et al., 2020), but also for its positive impact on mood and mental health (Singh et al., 2023). Despite these benefits, 45% of European individuals lead sedentary lifestyles (European Commission, 2022), a trend worsened by the shift toward office-based and online jobs that promote inactivity (Wilms et al., 2022). Given that people spend the majority of their waking hours at work, workplace-based physical activity interventions present an opportunity to boost activity levels and improve health (Rizzato et al., 2022). A recent systematic review (Abdin et al., 2018) highlighted that physical activity interventions in office-based settings can positively impact employees’ mental health and work-related outcomes. Specifically, employees who participated in workplace physical activity interventions reported reduced anxiety, depression, and stress symptoms, along with improvements in wellbeing, life satisfaction, productivity, and job performance. Workplace physical activity interventions can take various forms, including structured exercise programs, walking initiative, and mind–body practices such as Yoga or Tai Chi (Abdin et al., 2018; Latino et al., 2021; To et al., 2013).

Yoga is an ancient practice (whose name means “union” in Sanskrit) that sets its roots in the Indian culture and involves a combination of physical postures (i.e., asana), breathing techniques (i.e., pranayama), concentration, and meditation. Tai Chi is a martial art originating from China that combines slow body movements with deep breathing, meditation, and relaxation (National Center for Complementary and Integrative Health, 2023). These disciplines have demonstrated positive health effects, significantly impacting symptoms of depression, anxiety, stress, overall wellbeing, and quality of life (Cramer et al., 2018, 2013; Wang et al., 2014). Additionally, the practice of Yoga seems to support interpersonal functioning by improving assertiveness, as shown in a recent study (Jarry et al., 2017). Given their benefits, Yoga and Tai Chi practices have been integrated in workplace health promotion programs, where they have been proven effective in enhancing health and wellness, particularly by reducing work-related stress (Cocchiara et al., 2020; Della Valle et al., 2020).

Among various occupational sectors, educational settings are particularly known for their high demands, often leading to substantial workloads and exposing school and academic staff to significant stress (Lee et al., 2022; Watts and Robertson, 2011). Consequently, identifying effective interventions to reduce distress and enhance coping strategies in schools and universities is essential to support both individual wellbeing and institutional productivity. In this context, previous research has explored the beneficial effects of mind–body interventions (i.e., Yoga and Tai Chi) specifically within educational settings. Studies focusing on students (Cozzolino et al., 2022; Strehli et al., 2021) and teachers (Hwang et al., 2017; Latino et al., 2021) have shown that mindfulness training and yoga-based programs are effective in mitigating stress and improving psychological wellbeing and physical health. Despite these promising findings, only a few studies have specifically focused on employees in higher-level educational settings, such as academia or universities. For example, a study by Hartfiel et al. (2011) demonstrated that a 6-week Yoga program conducted in a British university was effective in improving mood and resilience in 48 academic employees. The Yoga group reported reduced anxiety, depression, confusion, fatigue and increased life satisfaction, sense of purpose, and self-confidence in stressful situations compared to the wait-list control group. In another study, Tamim et al. (2009) implemented a 12-week Tai Chi intervention with 52 female university employees. Though lacking a control group, the study found significant reductions in stress and improvements in psychological wellbeing and musculoskeletal fitness. However, these studies (Hartfiel et al., 2011; Tamim et al., 2009) had relatively small sample sizes and used a limited range of instruments to assess psychological wellbeing, which may not have fully captured the effects of Yoga or Tai Chi on specific dimensions of wellbeing. Despite these limitations, results of these studies are encouraging, especially considering that both Yoga and Tai Chi are safe, low-cost interventions that can be easily implemented in workplace settings (Cramer et al., 2018; Puerto Valencia et al., 2019).

These studies suggest that health promotion programs should be implemented in academia to improve employees’ overall health and foster better working conditions. Notably, recent research indicate that Italian academic staff perceive the university environment as highly stressful, contributing to significant levels of anxiety (Tontodimamma et al., 2024). In addition, various mental health issues have been reported among educational employees in Italy (Truzoli et al., 2021), indicating a widespread problem. Academic and school personnel may face specific work-related stressors, such as heavy workloads and responsibilities, job insecurity, perception of unfairness and inequality, lack of support, and strained relationships with colleagues, supervisors, or students (Tontodimamma et al., 2024; Truzoli et al., 2021).

This evidence emphasizes the importance of testing and implementing feasible intervention strategies to reduce the effects of work-related stress and improve employees’ health conditions in the Italian context. However, there is a notable lack of research on the impact and feasibility of mind–body interventions in the Italian academic context. To the best of our knowledge, only one study (Lazzeri et al., 2019) has examined the effects of a workplace health promotion project on risk behaviors among employees at an Italian university hospital. This study, however, focused solely on the impact of health promotion messages distributed throughout the workplace, encouraging health-protective behaviors to reduce the frequency of health risk behaviors. Lazzeri et al. (2019) did not employ a structured physical activity intervention, nor did they explore the potential benefits of mind–body interventions on employees’ psychological health. Additionally, although the study took place in an academic setting and included participants with varied roles, it focused exclusively on hospital personnel, omitting employees from other roles and settings. To address these gaps, our study aims to examine the effects of a structured mind–body intervention within the Italian academic context, encompassing employees across diverse roles and settings.

### 1.1 The current study

The purpose of the present study is to evaluate the impact of a structured Yoga/Tai Chi intervention in improving Italian university employees’ psychological wellbeing and perceived mental and physical health. More precisely, the present study aims to analyze the possible beneficial role that these mind–body programs might have on (i) anxiety, depressive, and stress symptoms; (ii) perceived physical and mental health; and (iii) assertive and cooperative behaviors among university employees. Furthermore, this research aimed to explore the impact of a single Tai Chi/Yoga session on Italian university employees’ state anxiety levels.

Based on previous studies (Chugh-Gupta et al., 2013; Hartfiel et al., 2011; Lee et al., 2009; Sharma and Haider, 2015; Tamim et al., 2009), we expect to observe lower levels of depressive, stress, and anxiety symptoms, including state anxiety, and higher levels of both perceived physical and mental health following the participation in Tai Chi and Yoga sessions in a sample of Italian university employees. Furthermore, we expect to observe an increase in the self-reported attitude toward assertive and cooperative behaviors following the practice of Tai Chi and Yoga programs, as these activities are expected to positively impact the psychological wellbeing and perceived mental and physical health of employees (Ilkhchi et al., 2011; Seçer et al., 2014).

In comparison to internationally published studies (Hartfiel et al., 2011; Tamim et al., 2009), our study aims to address a larger audience (i.e., in terms of sample size), investigate different constructs related to psychological wellbeing and perceived mental and physical health, and assess the acute effects of mind–body practices on state anxiety. Thereby, the findings of the present study have the potential not only to replicate international results but also to enrich understanding within the Italian context, providing valuable insights to inform workplace policies and best practices.

## 2 Materials and methods

### 2.1 Participants

**Figure 1:**
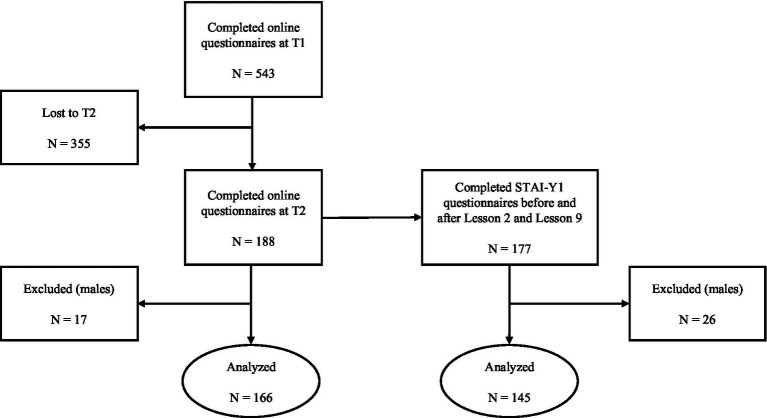
Flow diagram of participants through the Tai Chi/Yoga interventions.

A total of 543 employees (66 males) from the University of Padova participated free of charge in either Yoga or Tai Chi programs. Each program consisted of one-hour lessons of either Yoga or Tai Chi, held once or twice a week for a total of 10 sessions. Only a subsample of 188 participants (17 males) completed the self-report questionnaires both before (pre-test) and after (post-test) the Yoga or Tai Chi program. Participants who completed the program and those who dropped out from the program did not differ in pre-test scores (all *p*_s_ > 0.05; see Supplementary Materials). Due to the reduced number of male participants, only data from female participants were included in the analysis. Hence, the final sample comprised 166 female employees (*M*_age_ = 43.77, *SD*_age_ = 11.67). Regarding state anxiety, 177 employees (26 males) completed the STAI-Y1 before and after the second and second-to-last lessons of the Yoga/ Tai Chi program. Similarly, only data from female participants were considered, resulting in a final sample of 145 females (*M*_age_ = 45.23, *SD*_age_ = 11.43). Table 1 provides further information about participants’ socio-demographic characteristics. Figure 1 presents a flow diagram detailing the progression of participants through each stage of the study.

**Table 1:** Demographic characteristics of female participants that completed the questionnaires before and after the Tai Chi/Yoga program (*n* = 166) and that completed the STAI-Y1 before and after the second and second-to-last lessons of Tai Chi/Yoga program (*n* = 145).

	*n* = 166	*n* = 145
	Mean (SD) or *n* (%)	Mean (SD) or *n* (%)
**Age**	43.77 (11.67)	45.23 (11.43)
**Marital status**		
Single	45 (27.1)	36 (24.8)
With partner not cohabiting	0 (0)	0 (0)
Married/Cohabiting	112 (67.5)	96 (66.2)
Separated/Divorced	7 (4.2)	11 (7.6)
Widowed	2 (1.2)	2 (1.4)
**Years of education**	17.57 (3.40)	17.76 (2.84)
**Physical activity**		
No	68 (41.0)	61 (42.1)
Yes	98 (59.0)	84 (57.9)

### 2.2 Instruments

The socio-demographic information schedule was administered before the structured Yoga/Tai Chi intervention only (T1). All other assessments occurred at two time points: before the structured Yoga/Tai Chi intervention (T1) and after the structured Yoga/Tai Chi intervention (T2). Additionally, the state anxiety assessment occurred before and after the second and second-to-last lessons.

#### 2.2.1 Socio-demographic information schedule

A series of *ad-hoc* questions were posed to the participants to investigate their socio-demographic characteristics (e.g., age, gender, years of education, marital status).

#### 2.2.2 Somatic anxiety

Somatic anxiety was assessed using the Beck Anxiety Inventory (BAI; Beck et al., 1988; Sica et al., 2006), a 21-item measure evaluating the severity of somatic anxiety symptoms. Responses are rated on a 4-point Likert scale (from 0 “not at all” to 3 “severely”). Examples of symptoms investigated include being “Nervous,” “Unable to relax,” or having “Indigestion.” Higher scores indicate a greater severity of somatic anxiety. The Italian version of the BAI (Sica et al., 2006) showed good internal consistency (*α* = 0.89) and a good 30-day test–retest reliability (*r* = 0.62).

#### 2.2.3 Ruminative thoughts

Ruminative thoughts were evaluated using the Penn State Worry Questionnaire (PSWQ; Meyer et al., 1990; Morani et al., 1999) a 16-item instrument assessing the intensity, frequency, and uncontrollability of worry. Responses are scored on a 5-point Likert scale (from 1 “not at all typical of me” to 5 “very typical of me”). Examples of items include “My worries overwhelm me” and “Once I start worrying, I cannot stop.” A higher score represents a greater degree of pathological worry. The Italian version of the PSWQ (Morani et al., 1999) indicated good to excellent psychometric properties (e.g., internal consistency: *α* = 0.85).

#### 2.2.4 General distress

General distress was assessed using the Depression Anxiety Stress Scales-21 (DASS-21), a 21-item questionnaire (Bottesi et al., 2015; Lovibond and Lovibond, 1996) investigating depressive, anxious, and stress symptoms over the past week on a 4-point Likert scale (from 0 “did not apply to me at all” to 3 “applied to me very much”). Examples of questions include “I found it difficult to relax,” “I felt I was close to panic,” and “I felt that life was meaningless.” As suggested by Bottesi et al. (2015), we used the DASS-21 total score as a measure of “general distress,” where higher scores indicate greater distress symptoms. The total score of the Italian version of the DASS-21 (Bottesi et al., 2015) showed excellent internal consistency and good 2-week test–retest reliability (*α* = 0.90 and *r* = 0.74).

#### 2.2.5 Perceived health

The Short Form Health Survey-12 (SF-12; Apolone, 2001; Ware et al., 1996) was employed to evaluate perceived health. The SF-12 is the brief version of the SF-36, including 12 items. Responses are rated dichotomously (yes/no – e.g., “During the past 4 weeks have you accomplished less than you would like with your work or other regular activities as a result of your physical health?”) or on a Likert scale (3- or a 5-point – e.g., “Have you felt downhearted and blue?”). The SF-12 evaluates 8 dimensions of quality of life that can be summarized in 2 scores assessing physical (PCS) and mental health (MCS). These latter were considered for our analyses. Higher values suggest a greater perceived quality of life (QoL). The Italian version of the SF-12 has shown good psychometric properties (Apolone, 2001).

#### 2.2.6 General assertiveness

General assertiveness was assessed with the Scale for Interpersonal Behavior – short form version (SIB-r; Arrindell et al., 2002, 1984), a 25-item questionnaire collecting information about interpersonal behaviors (Arrindell et al., 2002, 1984). Participants indicated their answer on a 5-point Likert scale (from 1 “The situation does not cause you any discomfort/anxiety” to 5 “The situation causes you very strong discomfort/anxiety”). The SIB-r includes 4 subscales: Display of negative feelings (Negative assertion – e.g., “Refusing to lend something to a near acquittance”); Expression of and dealing with personal limitations (e.g., “Asking someone to show you the way”); Initiating assertiveness (e.g., “Giving your opinion to a person in authority”); Praising others and the ability to deal with compliments/praise of others (Positive assertion – e.g., “Telling someone that you like him/her”). In addition to the subscales, a total score of General Assertiveness can be computed. For the purposes of the current study, we focused only on the total score of general assertiveness. The Italian version of the SIB-r (Arrindell et al., 2002) showed good internal consistency for the total score (*α* = 0.90).

#### 2.2.7 Cooperativeness

The Cooperativeness Scale (Bonaiuto, 1997; Lu and Argyle, 1991) is a 33-item questionnaire measuring the attitude toward cooperative behaviors. This Italian scale was developed based on the original Cooperativeness Scale constructed by Lu and Argyle (1991). Responses are rated on a 7-point Likert scale (from 1 “totally agree” to 7 “totally disagree). It comprises 3 dimensions: “Benefits and gratifications of cooperation,” “Difficulties and disagreements in cooperation,” and “Competition and decision-making autonomy.” Examples of items include “Among friends there is often disagreement because everyone wants to do something different from the others” and “Before deciding, it is always preferable to consult colleagues.” The Italian version of the questionnaire (Bonaiuto, 1997) showed good internal consistency for the 3 dimensions, as well as good test–retest reliability (from *α* = 0.71 to *α* = 0.81; *r* = 0.83).

#### 2.2.8 State anxiety

We used the Y1 form of the State–Trait Anxiety Inventory (STAI-Y1; Pedrabissi and Santinello, 1989; Spielberger et al., 1983) to measure state anxiety levels of the participants. STAI-Y1 is composed of 20 items evaluating respondents’ level of anxiety “right now, at this moment.” Answers are given on a 4-point Likert scale (from 1 “not at all” to 4 “very much so”), with a higher score indicating greater anxiety. Examples of items include “I feel calm” and “I am tense.” The Italian version of the questionnaire (Pedrabissi and Santinello, 1989) showed good internal consistency levels (from *α* = 0.91 to *α* = 0.95).

### 2.3 Procedure

Before the opening of the Yoga and Tai Chi programs’ registration, a “Save the date” email was sent to all employees of the University of Padova (including technical-administrative staff, teaching, and research staff, such as research fellows and Ph.D. students). The email informed them of the opportunity to participate, free of charge, in a 10-h Tai Chi or Yoga programs and provided details about the registration date and time. Approximately 2 weeks before the programs began, a second email was sent to all employees announcing the opening of registration and containing the enrollment form.

The research project was then presented on the first day of Yoga/Tai Chi lessons. All attendees of the first lesson received an email invitation to participate in the research at the conclusion of the class, along with additional information and a link to voluntarily complete an online survey available via Google Forms. After giving their informed consent, participants provided socio-demographic information and, both before and after the 10-session Yoga/Tai Chi programs, completed the BAI, PSWQ, DASS-21, SF-12, SIB-r, and Cooperativeness Scale questionnaires. The STAI-Y1 questionnaire was administered in paper and pencil format at the beginning and the end of the second and second-to-last lessons. At the end of the last lesson, participants received an email inviting them to complete the same online questionnaires that were administered after the first class.

**Figure 2:**
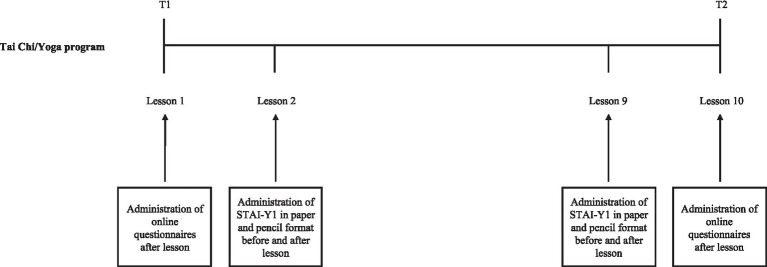
Study procedure.

Participants did not receive any kind of compensation for participating in the research project, and they were fully informed about their possibility to withdraw from the study at any time, without penalty. The study procedure is illustrated in Figure 2.

The study was conducted in accordance with the Declaration of Helsinki and was approved by the relevant departmental ethics committee at the School of Psychology, University of Padova.

### 2.4 Yoga and Tai Chi interventions

The Yoga intervention consisted of sessions of approximately 1 h and 15 min each, held once a week after working hours, either outside or inside the University facilities. The classes focused on teaching simple asanas to improve physical wellbeing, breathing techniques to develop emotional and mental balance, and relaxation techniques to reduce stress and foster overall emotional wellbeing, while shifting mental focus to internal sensations (Büssing et al., 2012; Evans et al., 2009). Each Yoga session comprised 10 min of meditation to focus on the present moment, 10 min of gradual exercises for the spine, 20 min of dynamic muscle stretching, 20 min of breathing exercises, and 15 min of final relaxation.

The Tai Chi intervention consisted of classes of approximately 1 h each, held twice a week before working hours or during the lunch break, either outside or inside the University facilities. The program focused on the study of the Water Taiji style founded by Master Wang Zhuanghong and included the sequence of ‘Ba Duan Jin’ along with the first 7 movements of ‘Shi Pa Luohan Gong’. These sequences promote the release of internal energy to improve vitality and alleviate body tension (Lan et al., 2013; Zou et al., 2018a). Additionally, the program incorporated relaxation techniques and an introduction to Vipassana meditation in the Theravada tradition, following the Mahasi Method (Mahasi, 2016). Each Tai Chi session included 10 min of warm-up to release tension and prepare the body for movement, 10 min of basic exercises to develop Tai Chi fundamentals, 20 min of posture exercises and study of Water Taiji style, 10 min of relaxation, and 10 min of Vipassana meditation.

### 2.5 Statistical analysis

All statistical analyses were conducted using IBM SPSS Statistics software (Version 28.0.1; IBM Corp., Armonk, NY, USA). Paired t-tests were performed to compare participants’ psychological wellbeing and perceived mental and physical health before and after the Yoga and Tai Chi programs, and to compare state anxiety levels before and after the second and second-to-last lessons. In case of a significant difference in the questionnaires’ scores before and after the programs, Cohen’s *d* was calculated to assess the magnitude of the effect. Results with a *p* < 0.05 were considered statistically significant.

## 3 Results

Paired t-tests were used to compare participants’ psychological wellbeing and perceived mental and physical health before and after the Yoga and Tai Chi programs. Results showed a significant difference between pre and post program for the PSWQ (*p* = 0.007), the BAI (*p* < 0.001), and the mental component of the SF-12 (*p* = 0.038). This indicates that there was a reduction in ruminative thoughts (*M_pre_* = 51.02, *SD_pre_* = 12.66; *M_post_* = 49.21, *SD_post_* = 12.01) and somatic anxiety (*M_pre_* = 9.92, *SD_pre_* = 6.75; *M_post_* = 8.33, *SD_post_* = 5.56), and improvements in perceived mental health (*M_pre_* = 41.89, *SD_pre_* = 9.65; *M_post_* = 43.27, *SD_post_* = 9.50) following Yoga and Tai Chi classes. No significant differences were found for the physical component of the SF-12 (*p* = 0.296), the DASS-21 (*p* = 0.084), SIB-r (*p* = 0.508), and the Cooperativeness Scale (Benefits and gratifications of cooperation: *p* = 0.427; Difficulties and disagreements in cooperation: *p* = 0.138; Competition and decision-making autonomy: *p* = 0.638). Table 2 details the results regarding the scores obtained at the questionnaire pre and post Tai Chi/Yoga programs.

**Table 2:** Scores at the questionnaires before and after Tai Chi/Yoga interventions.

	Pre	Post				
	Mean (SD)	Mean (SD)	*t*	*df*	*p*	Cohen’s *d*
**Ruminative thoughts (PSWQ)**	51.02 (12.66)	49.21 (12.01)	2.714	165	0.007	0.211
**Somatic anxiety (BAI)**	9.92 (6.75)	8.33 (5.56)	3.777	165	< 0.001	0.293
**General distress (DASS-21)**	14.99 (9.98)	13.65 (9.71)	1.741	150	0.084	/
**Perceived health (SF-12)**						
Mental health (MCS)	41.89 (9.65)	43.27 (9.50)	−2.095	164	0.038	−0.163
Physical health (PCS)	51.84 (7.11)	52.37 (6.78)	1.049	164	0.296	/
**General assertiveness (SIB-r)**	64.69 (17.14)	64.12 (17.40)	0.663	165	0.508	/
**Cooperativeness (Cooperativeness scale)**						
Benefits and gratifications of cooperation	57.92 (9.73)	58.38 (9.56)	−0.796	150	0.427	/
Difficulties and disagreements in cooperation	52.60 (10.50)	53.52 (10.20)	−1.492	150	0.138	/
Competition and decision-making autonomy	47.60 (6.86)	47.30 (6.89)	0.471	150	0.638	/

BAI, back anxiety inventory; DASS-21, depression anxiety stress scales-21; MCS, mental component score; PCS, physical component score; PSWQ, Penn State worry questionnaire; SF-12, short form health survey-12; SIB-r, scale for interpersonal behavior – short form version.

Finally, we found a significant reduction in state anxiety after 1 h of Yoga/Tai Chi, both at the second (*p* < 0.001; *M_pre_* = 40.60, *SD_pre_* = 9.61; *M_post_* = 30.11, *SD_post_* = 7.15) and second-to-last sessions (*p* < 0.001; *M_pre_* = 40.61, *SD_pre_* = 9.85; *M_post_* = 29.83, *SD*_post_ = 7.44). These results are presented in detail in Table 3.

**Table 3:** Stat-trait anxiety inventory – form Y1 (STAI-Y1) scores before and after second and second-to-last Tai Chi/ Yoga lessons.

	Pre	Post				
	Mean (SD)	Mean (SD)	*t*	*df*	*p*	Cohen’s *d*
Second lesson	40.60 (9.61)	30.11 (7.15)	15.749	144	< 0.001	1.308
Second-to-last lesson	40.61 (9.85)	29.83 (7.44)	14.504	144	< 0.001	1.205

## 4 Discussion

Occupational settings are widely recognized as major contributors to employee distress, significantly influencing overall health (Hilmiana and Alviani, 2023; Martins et al., 2023). Similarly, over the past few decades, academic institutions have witnessed an increase in stress levels and a general decline in employee wellbeing (Tontodimamma et al., 2024; Truzoli et al., 2021). Academic life, in particular, presents several ongoing challenges for staff members, which, when combined with heavier workloads, might create a stressful environment (Tontodimamma et al., 2024; Truzoli et al., 2021). Workplace physical activity interventions may serve as effective tools for improving employees’ mental health and job-related outcomes (Abdin et al., 2018). In this context, we aimed to investigate the potential positive effects of 10-h Yoga and Tai Chi interventions on the psychological wellbeing and perceived mental and physical health of female university employees.

According to our results, participation in Tai Chi or Yoga programs plays a significant beneficial role in enhancing psychological wellbeing among female university employees. Specifically, Yoga and Tai Chi programs significantly improved participants’ psychological wellbeing by reducing the frequency of ruminative thoughts and somatic anxiety symptoms, as evidenced by the lower scores on the PSWQ and the BAI after the Tai Chi/Yoga programs. These findings align with previous research showing that Yoga and Tai Chi practice effectively reduce worry among college students (Eastman-Mueller et al., 2013) and somatic symptoms in both clinical and academic settings (Gabriel et al., 2018; Mulcahy et al., 2020). Additionally, our results are consistent with research conducted with academic employees, where Yoga-based interventions have been shown to improve mood states (Hartfiel et al., 2011). Our study contributes the growing body of evidence demonstrating that both Yoga and Tai Chi mitigate adverse psychological outcomes, such as rumination and anxiety, which are frequently exacerbated by workplace stressors. Both practices include meditative techniques that enhance interoceptive awareness, encouraging individuals to focus on present bodily sensations. This shift in attention provides an alternative focus to negative cognitive processes, thereby reducing ruminative thinking (Gibson, 2019).

The Tai Chi/Yoga programs also significantly improved participants’ perceived mental health, as evidenced by higher scores on the psychological dimension of the SF-12. This result aligns with previous studies reporting that general health status often improves following Tai Chi and Yoga interventions (Benavidez and Hart, 2017; Wang et al., 2014). However, no significant effects were observed for the dimension of perceived physical health. Previous studies have reported mixed outcomes regarding the impact of mind–body interventions on perceived physical and mental health. While some studies have shown improvements in both dimensions (Lee et al., 2009), others have found benefits limited to the mental component (La Torre et al., 2020; Lindahl et al., 2016), or the physical component alone (Wang et al., 2020). The lack of improvements in perceived physical health in our participants might be explained by the short duration of the Tai Chi/Yoga programs, which consisted of only 10 total hours of practice. Other factors, such as exercise intensity, session frequency, participants’ fitness levels, or baseline physical status, could also contribute to this outcome. For instance, the study by Lee et al. (2009), which found improvements in both physical and mental health, was based on a program that lasted 26 weeks and comprised 1-h session of Tai Chi three times a week. Conversely, other authors (La Torre et al., 2020) observed mental health benefits but no changes in physical health after an 8-h yoga and mindfulness intervention. These findings suggest that mental health may improve more rapidly after mind–body interventions, while significant physical health benefits might require longer program durations, increased session frequency, or both. Additionally, baseline individual differences in physical condition may influence the perception of changes in physical health (Caramenti and Castiglioni, 2022; Sani et al., 2016).

Existing research suggests that Yoga and Tai Chi interventions can alleviate perceived general stress levels (de Manincor et al., 2016; Wang et al., 2014; Zou et al., 2018b). However, our study did not find significant effects of these interventions on general distress. This finding contrasts with previous findings among female academic workers, where Tai Chi effectively reduced perceived stress (Tamim et al., 2009). A plausible explanation for this outcome may be attributed to the participants’ low general distress scores at the start of the program. This aligns with a recent workplace satisfaction survey conducted in leading Italian organizations, which identified the University of Padova as offering a positive work environment (Sottocornola, 2023). Indeed, mind–body interventions often yield greater benefits when individuals present with higher baseline levels of distress (Hofmann et al., 2016). Nevertheless, this discrepancy highlights the need for further investigation into the factors that influence the effectiveness of mind–body interventions on general distress within specific populations and contexts.

Furthermore, no significant improvements in assertiveness or cooperativeness were observed among female university employees following the Yoga/Tai Chi programs. This may be attributed to the nature of Yoga and Tai Chi practices, which do not inherently involve interaction or socialization among participants, factors considered essential for the development of assertive and cooperative behaviors (Johnson et al., 2014; Speed et al., 2018). Although improvements in psychological well-being could theoretically enhance interpersonal skills (Abdelaziz et al., 2020; Kokko et al., 2013), other factors likely contribute to their development. According to the literature, cooperative behavior is shaped by a combination of personal, relational, and task-related factors (Sommet et al., 2023). Similarly, difficulties in assertiveness may not solely result from psychological factors, such as anxiety, but may also stem from behavioral deficits, including inadequate communication skills (Speed et al., 2018). Consequently, reduced anxiety does not necessarily translate into more assertive behaviors if the necessary skills are lacking (Speed et al., 2018). Incorporating group activities into mind–body interventions could address this gap by fostering opportunities for interaction, enabling participants to practice assertiveness and cooperativeness in appropriate social contexts (Johnson et al., 2014; Speed et al., 2018). This approach may enhance the potential of these programs to support the development of interpersonal skills.

The present study showed that even a single Yoga/Tai Chi class effectively reduces state anxiety, as evidenced by lower state anxiety levels at the end of a class compared to the beginning. Previous research has similarly reported reductions in state anxiety following participation in mind–body interventions (Chugh-Gupta et al., 2013; Lin et al., 2024; Sharma and Haider, 2015). However, most studies suggest that such reductions typically occur after completing a series of classes (La Torre et al., 2020; Ranjita et al., 2016). Our findings contribute to this body of literature by showing that even a single session of Yoga or Tai Chi can significantly reduce state anxiety levels among university employees. This underscores the value of these practices as tools for managing anxiety in high-stress situations, such as specific job-related activities (Cheng and McCarthy, 2018). These findings have important implications not only for individual well-being but also for organizational outcomes, as lower levels of anxiety are known to enhance workplace performance (Powell et al., 2018).

There are important limitations to consider that may restrict the generalizability of the results in the present study. First, the study did not include a control group due to practical and accessibility constraints. As a result, the effects of the Tai Chi and Yoga programs were examined only within the intervention group, limiting the ability to draw definitive conclusions about the specific impact of these interventions. Additionally, male participants were excluded from the analyses because of their low representation in the sample, meaning the findings presented here are specific to the female participants.

Despite these limitations, the study’s naturalistic setting offers ecologically valid insights into how mind–body programs may affect female university employees in real-life workplace contexts. Future studies should aim for a larger, more balanced sample that includes both male and female employees to explore the potential benefits of mind–body exercises across genders. Furthermore, future studies should consider including a control group, such as a waiting list or an active control group, to properly estimate the effects of Tai Chi and Yoga interventions on the examined psychological variables, excluding potential confounders, and still providing the employees with the opportunity to engage in wellness programs.

The results of this study have important practical implications for both organizations and individuals, suggesting that incorporating Tai Chi and Yoga practices into the workplace can improve psychological wellbeing and perceived mental health among female workers, ultimately fostering a healthier and more positive work environment. This is encouraging, considering that Tai Chi and Yoga interventions are cost-effective and easy to implement in various work settings. Furthermore, the findings indicate that employees can use these practices to quickly achieve a sense of calm and improve focus on work tasks. The rapid psychological benefits of mind–body exercises may motivate individuals to engage in them regularly, laying the foundation for a healthier and more balanced lifestyle.

## 5 Conclusion

In conclusion, this study provides valuable insights to the growing body of research on the benefits of mind–body interventions for psychological wellbeing and mental health, particularly in the Italian academic context.

Our findings demonstrate that Yoga and Tai Chi programs effectively enhance psychological well-being by reducing ruminative thoughts and somatic anxiety, while improving perceived mental health among female university employees. The study also highlights that even a single Tai Chi or Yoga session can significantly reduce state anxiety, emphasizing their immediate benefits and the potential value of incorporating these practices into the workplace for short-term stress relief.

By providing empirical evidence of the benefits of Tai Chi and Yoga, this study lays the foundation for further research on the applicability and scalability of these interventions in academic settings. Future research should address the limitations of the present study and explore other work environments to deepen our understanding of the potential benefits of mind–body interventions across different populations and contexts.

## Data Availability

The raw data supporting the conclusions of this article will be made available by the authors, without undue reservation.
