# Supplementary material for: Promoting workplace psychological wellbeing through Yoga and Tai Chi classes in female university employees
Source: Front Psychol. 2024 Dec 12;15:1502426. doi: 10.3389/fpsyg.2024.1502426 (PMC11670327; doi:10.3389/fpsyg.2024.1502426)
Supplement: Supplementary file 1 [file Table_1.docx]

Supplementary Material

# Supplementary Tables

**Supplementary Table 1.** Differences in baseline scores at questionnaires between participants who completed and those who did not complete the Tai Chi/Yoga programs

|  | Not Completed | Completed |  |  |  |
| --- | --- | --- | --- | --- | --- |
|  | Mean (SD) | Mean (SD) | *t* | *df* | *p* |
| **Ruminative thoughts (PSWQ)** | 49.57 (13.31) | 51.02 (12.66) | -1.148 | 461 | 0.252 |
| **Somatic Anxiety (BAI)** | 10.04 (7.26) | 9.92 (6.74) | 0.168 | 461 | 0.867 |
| **General distress (DASS-21)** | 16.16 (10.70) | 14.99 (9.98) | 1.098 | 420 | 0.273 |
| **Perceived health (SF-12)** |  |  |  |  |  |
| *Mental health (MCS)* | 40.28 (9.98) | 41.85 (9.63) | -1.647 | 461 | 0.100 |
| *Physical health (PCS)* | 52.12 (7.40) | 51.85 (7.09) | 0.383 | 461 | 0.702 |
| **General assertiveness (SIB-r)** | 63.90 (17.19) | 64.70 (17.14) | -0.473 | 461 | 0.637 |
| **Cooperativeness (Cooperativeness scale)** |  |  |  |  |  |
| *Benefits and gratifications of cooperation* | 57.15 (9.54) | 57.92 (9.72) | -0.789 | 420 | 0.431 |
| *Difficulties and disagreements in cooperation* | 52.69 (11.38) | 52.60 (10.49) | 0.087 | 420 | 0.931 |
| *Competition and decision-making autonomy* | 47.37 (6.67) | 47.60 (6.86) | -0.341 | 420 | 0.733 |
| *Notes*. BAI = Back Anxiety Inventory; DASS-21 = Depression Anxiety Stress Scales-21; MCS = Mental Component Score; PCS = Physical Component Score; PSWQ = Penn State Worry Questionnaire; SF-12 = Short Form Health Survey-12; SIB-r = Scale for Interpersonal Behaviour – short form version. | | | | | |
